# A Novel Crosstalk Suppression Method of the 2-D Networked Resistive Sensor Array

**DOI:** 10.3390/s140712816

**Published:** 2014-07-18

**Authors:** Jianfeng Wu, Lei Wang, Jianqing Li, Aiguo Song

**Affiliations:** 1 School of Instrument Science and Engineering, Southeast University, Nanjing 210096, China; E-Mails: ljq@seu.edu.cn (J.L.); a.g.song@seu.edu.cn (A.S.); 2 School of Automation, Nanjing Institute of Technology, Nanjing 211167, China

**Keywords:** 2-D resistive sensor array, measurement error, crosstalk suppression method, Improved Isolated Drive Feedback Circuit with Compensation (IIDFCC)

## Abstract

The 2-D resistive sensor array in the row–column fashion suffered from the crosstalk problem for parasitic parallel paths. Firstly, we proposed an Improved Isolated Drive Feedback Circuit with Compensation (IIDFCC) based on the voltage feedback method to suppress the crosstalk. In this method, a compensated resistor was specially used to reduce the crosstalk caused by the column multiplexer resistors and the adjacent row elements. Then, a mathematical equivalent resistance expression of the element being tested (EBT) of this circuit was analytically derived and verified by the circuit simulations. The simulation results show that the measurement method can greatly reduce the influence on the EBT caused by parasitic parallel paths for the multiplexers' channel resistor and the adjacent elements.

## 1. Introduction

Miniaturized and integrated resistive sensor arrays were important for sensing applications, such as tactile sensing [1–8], temperature sensing [8,9], light sensing [10,11], photoconductive image sensing [12], olfactory sensing [13], *etc*. Speeter [1] reported a flexible sensing system with 16 × 16 resistive taxels and achieved a sampling rate of more than 60 Hz. Castellanos-Ramos *et al.* [3] reported a 16 × 16 tactile sensor array based on conductive polymers with screen-printing technology. Fernando *et al.* [4] realized and compared three circuits of networked piezoresistive sensor arrays based on standard microcontrollers, programmable systems on chip and field programmable gate arrays. Fernando *et al.* [5] designed a 16 × 9 force sensor patch to cover large areas of robots and machines for interacting with human beings. Zhang *et al.* [6] reported a 3 × 3 thin tactile force sensor array based on conductive rubber. Kim *et al.* [7] reported a new concept of a flexible tactile sensor array capable of sensing contact force and position with high performance and high spatial resolution. Yang *et al.* [8] designed a 32 × 32 flexible array using PI-copper films within a 160 mm × 160 mm temperature and tactile sensing area, which could be used to recognize big size objects with different shape. Wu *et al.* [9] designed an 8 × 16 networked array of resistive temperature tactile device within a 16 mm × 32 mm sensing area. Saxena *et al.* designed a 16 × 16 bolometer array of IR detector [10,11] and a 16 × 16 imaging array of light dependent resistor [12]. Beccherelli *et al.* [13] reported a polymeric chemical sensor array consisting of 65,536 sensors comprising tens of different types. Discrete resistive sensors with different spatial resolution were widely used in these sensor arrays.

For accessing all *M* × *N* elements individually in these resistive sensor arrays with the tradition methods, *M* × *N* × 2 connection lines were needed. For reducing the number of connection lines, many circuits of networked sensor arrays were proposed with all resistive elements having one end connected to a row line and the other end to a column line [1–13]. Thus the interconnect line number of *M* × *N* resistive array is *M* + *N*. However, the measurement accuracy of the element being tested (termed as EBT here after) was interfered [14] with the crosstalk effect for parasitic parallel paths in these resistive sensor arrays with the row-column fashion. Many methods have been proposed in literatures such as the voltage feedback methods [1,9], the zero potential methods [3–8,10–12], and the inserting diode method [2], *etc*. In these methods, the zero potential methods and the voltage feedback methods were applied widely. Regarding circuit simulations with the PSpice, D'Alessio [14] evaluated the scanning circuits of piezoresistive sensors arrays and gave several guidelines for using these circuits. Regarding mathematical analysis, Saxena [10–12,15] proposed a mathematical expression of the crosstalk among all of the elements introduced due to the interconnection overloading and then verified them in the circuit simulations with the CADENCE-SPECTRE or the PSpice. Liu *et al.* [16] analyzed the measurement errors of the voltage feedback method by the PSpice program simulation. Wu *et al.* [17] presented a mathematical analysis of the voltage expression for the measurement output of the EBT in the networked resistive sensor array. Wu *et al.* [18] also presented a mathematical expression of the effective equivalent resistance of the EBT in the Isolated Drive Feedback Circuit. For suppressing the crosstalk or reducing the total current drawn from the buffer in these methods, complexity circuits with many op-amps and multiplexers were used. Fernando *et al.* [5–7,19,20] suppressed the crosstalk caused by the adjacent row or column elements with large number of op-amps using part virtual ground (PVG) technique. D'Alessio *et al.* [3,4,8,14] suppressed the crosstalk caused by the adjacent elements and the multiplexers with larger number of op-amps using full virtual ground (FVG) technique. In these methods, the measurement accuracy of the EBT suffered from the interferences of some parasitic parameters, such as the different offset and bias of the op-amps, and the different on resistances of the mux [14]. Thus the crosstalk suppression method with less complexity can improve the measurement accuracy of the EBT. Wu *et al.* [21] suppressed the crosstalk caused by the adjacent column elements with a special feedback voltage using only one op-amp. However, in the method of [21], the adjacent row elements and the column multiplexers still significantly interfered over the crosstalk. Thus, better crosstalk suppression methods with less complexity and cost are desired.

For this purpose, we present a novel crosstalk suppression method with less complexity and cost for the 2-D networked resistive sensor arrays in the row-column fashion. This paper begins with an overview of the application fields and the measurement methods in the 2-D networked resistive sensor arrays. Secondly, a novel crosstalk suppression method will be proposed and its mathematical equivalent resistance expression of the element being tested will be analytically derived. Then simulations will be implemented to evaluate this method with different parameters such as the measurement range of the EBT, the multiplexers' resistor, and the array size of 2-D networked resistive sensor arrays. Finally, the results of experiments will be analyzed and conclusions for the method will be given.

## 2. Theoretical Analysis of Our Crosstalk Suppression Method

**Figure 1.**
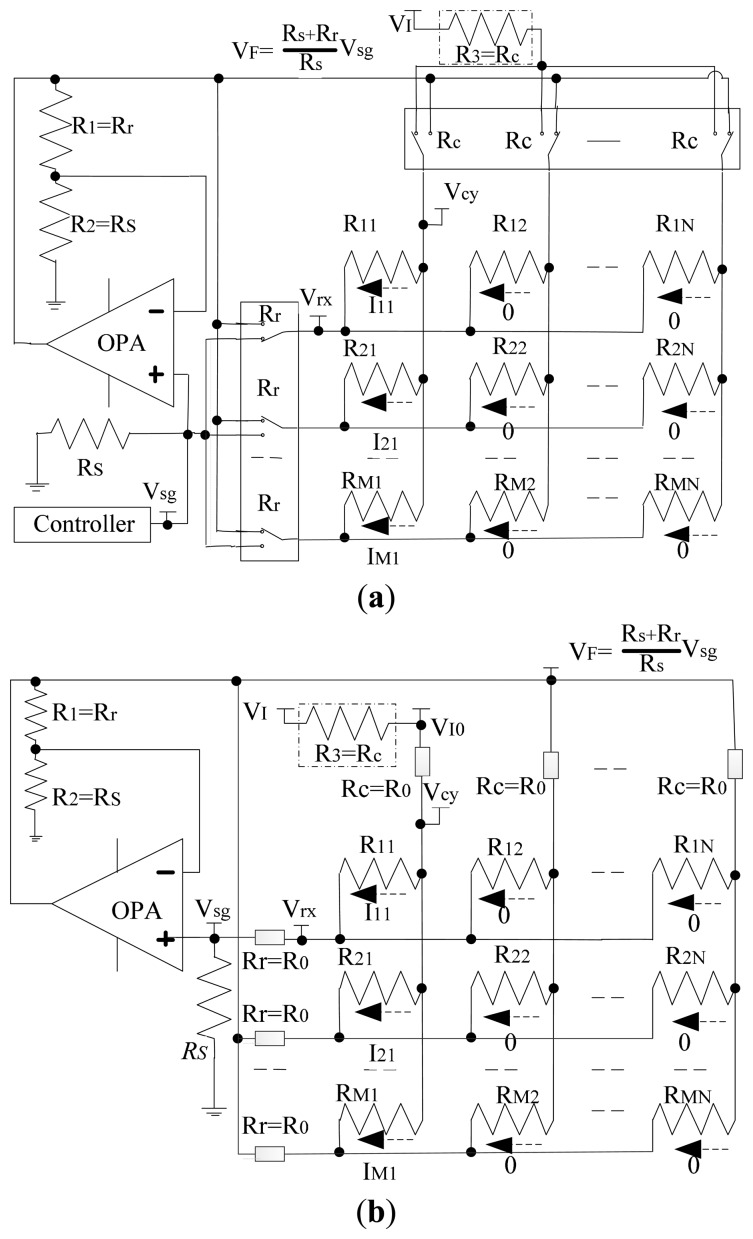
(**a**) Improved Isolated Drive Feedback Circuit with Compensation (IIDFCC) (Circuit A); (**b**) IIDFCC with the multiplexer's resistors (Circuit B).

In the literature [9,12,16,21], voltage feedback methods were proposed with only one op-amp. So the offset and the bias of different op-amps were reduced. In the Isolated Drive Feedback Circuit (IDFC) [9], the resistance value of the EBT was measured individually. In the Improved Isolated Drive Feedback Circuit (IIDFC) [21], a special feedback unit was adopted for reducing the influence of the column adjacent elements, and then the feedback voltage (*V_F_*) was equal to the voltage on the scanning row (*V_rx_*). Thus the currents of the column adjacent elements were approximated to zero, and the column adjacent elements and the channelresistors of the row multiplexers (*R_r_*) had less influence on the EBT. However, the row adjacent elements and the channel resistors of the column multiplexers (*R_c_*) still affected the measurement accuracy of the EBT. To have individual access of all resistors with a good measurement performance in the 2-D networked resistive sensor arrays, we propose using a novel crosstalk suppression method, which is based on the Improved Isolated Drive Feedback Circuit with Compensation (IIDFCC) of the column multiplexer resistor. We add another resistor (*R_3_* = *R_c_* as shown in the virtual box of the Circuit A in Figure 1a) to sample the current between the input voltage (*V_I_*) and the scanning column line. The resistance value of the scanning multiplex switch can be considered as a constant under the condition that the small tested current causes very little resistance change. Thus, Circuit A can be simplified to Circuit B as shown in Figure 1b where *R_11_* is the element being tested.

If the column multiplexer is ideal without leak current and *R_3_* is equal to *R_c_*, the voltage of the scanning column line can be expressed as Equation (1):

(1) $$V_{cy}=2V_{I0}-V_{I}$$

From IIDFC [21], *V_rx_* is equal to *V_F_*, so the current on the EBT is equal to the current on the sample resistor (*R_s_*). Thus the equivalent resistance value of the EBT can be expressed as Equation (2):

(2) $$R_{xy}=\frac{V_{cy}-V_{rx}}{V_{F}}(R_{s}+R_{r})=\frac{2V_{I0}-V_{I}-V_{F}}{V_{F}}(R_{s}+R_{r})$$

As *V_I_, R_s_* and *R_r_* are known, *V_I_*_0_ and *V_F_* can be measured by ADC, so the equivalent resistance value (*R_xy_*) of the EBT can be acquired.

## 3. Simulation Experiments and Discussion

Because the measuring temperature range of the small tactile device [9] was 10–50 °C, the resistance value of each sensitive element (NTC-103F950 thermistor of the SinoChip Electronic Co., LTD) was in the range[*R_min_, R_max_*], where *R_min_* = 3.6kΩ, and *R_max_* = 20 kΩ. The resistance value of the actual multiplexers was usually nonzero, and it could be 0.35–250 Ω [17]. To emulate the performance of our method, we simulated the 2-D networked resistive sensor array of the Circuit B with NI Multisim 12.

### 3.1. The R_xy_ Range Effect Simulation

**Figure 2.**
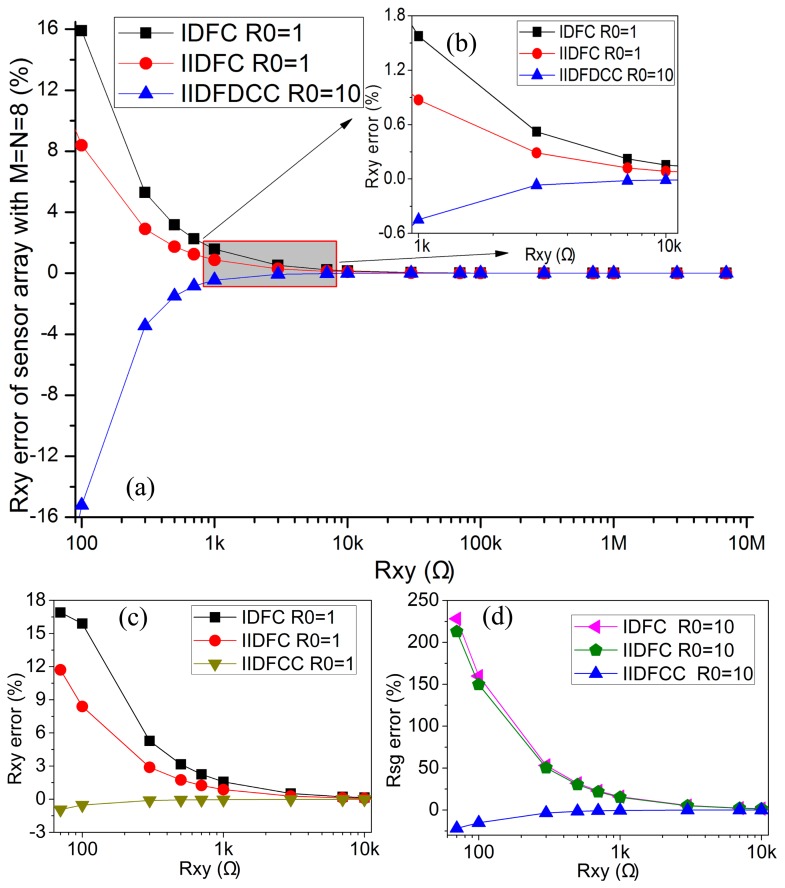
(**a**) The effect of *R_xy_* range on the *R_xy_* errors in IDFC (*R_0_* = 1 Ω), IIDFC (*R_0_* = 1 Ω), IIDFCC (*R_0_* = 10 Ω) where *M* = *N* = 8 and *R_xy_* ∈ (100 Ω–7 MΩ); (**b**) The effect of *R_xy_* range on the *R_xy_* errors in IDFC (*R_0_*= 1 Ω), IIDFC (*R_0_* = 1 Ω), IIDFCC (*R_0_* = 10 Ω) where *M* = *N* = 8 and *R_xy_* ∈ (1 kΩ–10 kΩ); (**c**) The effect of *R_xy_* range on the *R_xy_* errors in IDFC, IIDFC, IIDFCC where *M* = *N* = 8, *R_0_* = 1 Ω and *R_xy_* ∈ (100 Ω–10 kΩ); (**d**) The effect of *R_xy_* range on the *R_xy_* errors in IDFC, IIDFC, IIDFCC where *M* = *N* = 8, *R_0_* = 10 Ω and *R_xy_*∈ (100 Ω–10 kΩ).

When *R_0_* was fixed, varied range of *R_xy_* also affected the performance of the 2-D networked resistive sensor arrays. In the simulation, *M* = *N* = 8, *R_0_* = 1 Ω or *R_0_* = 10 Ω, all elements in sensor arrays and the sample resistor could vary synchronously with the resistance value in 100 Ω–7 MΩ. The simulation results of IDFC, IIDFC, and IIDFCC with NI Multisim 12 were shown in Figure 2.

From Figure 2, we found that IIDFCC had the minimum *R_xy_* errors with the resistance value in the wide range of 100 Ω–7 MΩ among three kinds of circuits. While *R_xy_* ≥ 1000Ω, IIDFCC with *R_0_* = 10 Ω still had a better performance than IIDFC and IDFC with *R_0_* = 1 Ω. Thus, IIDFCC has the best performance among three kinds of circuits in a wide range of *R_xy_*.

### 3.2. The Multiplexers' Channel Resistors (R_0_) Effect Simulation

**Figure 3.**
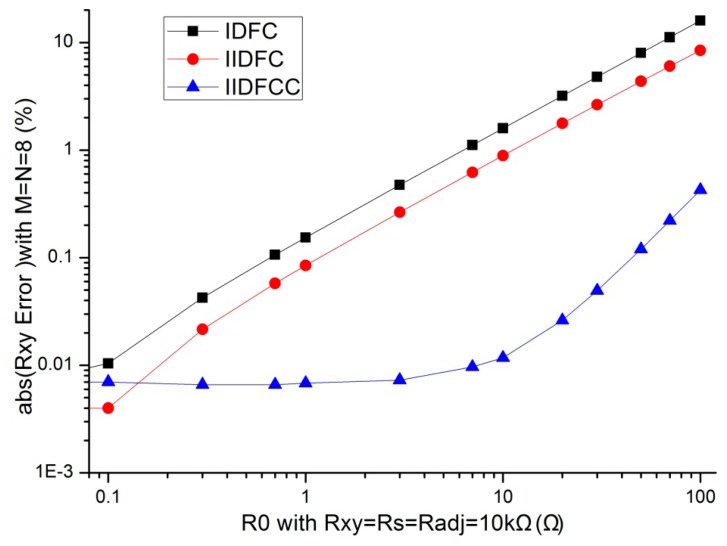
*R*_0_ effect on the *R_xy_* errors where *R*_0_ ∈ 0.10–100Ω.

In evaluating the effect of the multiplexers' column channel resistors (*R*_c_ = *R*_0_) and the multiplexers' row channel resistors (*R*_r_ = *R*_0_) in our method, the other parameters were set to constant values. If *R*_0_ is zero, *R_xy_* can be easily calculated. However, in an actual multiplexer, *R_0_* is nonzero. We fixed *M* = *N* = 8 and the resistance value of the sample resistor and all elements in resistive sensor array at 10 kΩ, IDFC, IIDFC, and IIDFCC were simulated with NI Multisim12 with *R_0_* varying in 0.10 Ω–100 Ω. The simulation results of measurement errors are shown in Figure 3.

From Figure 3, we found that, with the increasing of *R_0_* in this simulation, the measurement values of IDFC and IIDFC were larger than the actual value whereas the measurement values of IIDFCC were less than the actual value; the *R_xy_* errors of IDFC and these of IIDFC had a positive coefficient whereas the *R_xy_* errors of IIDFCC had a negative coefficient. From Figure 3, we found that the absolute measurement errors of these three circuits increased with the increasing of the multiplexer's channel resistance value. When *R_0_* was varied in 0.3Ω–100 Ω, the absolute measurement errors of IIDFCC were smaller than these of the other two circuits. Thus with our method, the *R_xy_* error caused by the multiplexers' channel resistors can be reduced greatly. Furthermore, multiplexers with bigger channel resistance can be used in the 2-D networked resistive sensor arrays.

### 3.3. The Array Size Effect Simulation

**Figure 4.**
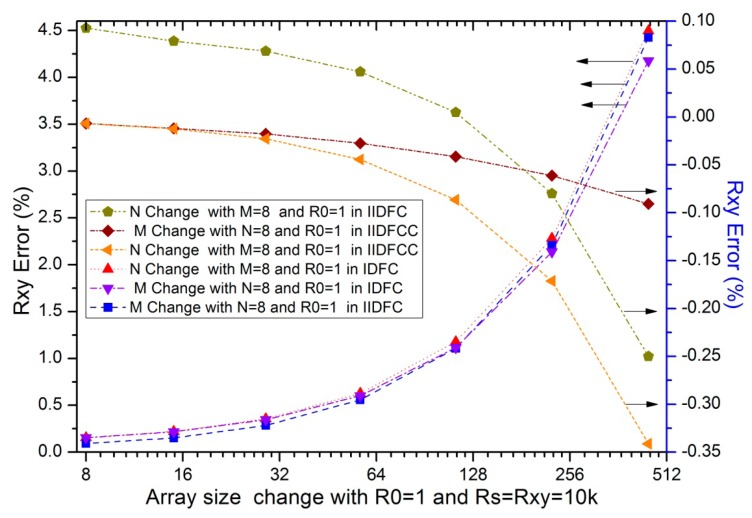
The array size effect on the *R_xy_* errors in three circuits where *R_0_* = 1 Ω and *R_xy_* = 10 kΩ.

Parameters of the array size such as the row number (*M*) and the column number (*N*) were proved that it had approximated influences on the performance of the 2-D networked resistive sensor arrays [17]. The influence of the column number had been significantly reduced with large number of op-amps using virtual ground technique [19,20] and with one op-amp using IIDFC [21]. In simulations, we fixed some parameters including the resistance value of the sample resistor and all elements in resistive sensor array at 10 kΩ, *M* or *N* at 8, *R_0_* at 1Ω, *N* or *M* was one of 8, 15, 29, 57, 113, 225, and 449. The simulation results of the array size effect of IDFC, IIDFC, and IIDFCC were shown in Figure 4.

As to the column number and the row number in IDFC and IIDFC, we found in Figure 4 that at least one parameter had a significant influence on their *R_xy_* errors; with the increase of the column number or the row number in IIDFCC, we found that the absolute *R_xy_* errors were reduced significantly compared with the other two circuits and the coefficient of its *R_xy_* errors was negative. Thus, the influence of the array size on the *R_xy_* error has been reduced greatly with our method.

### 3.4. The Adjacent Elements Effect Simulation

**Figure 5.**
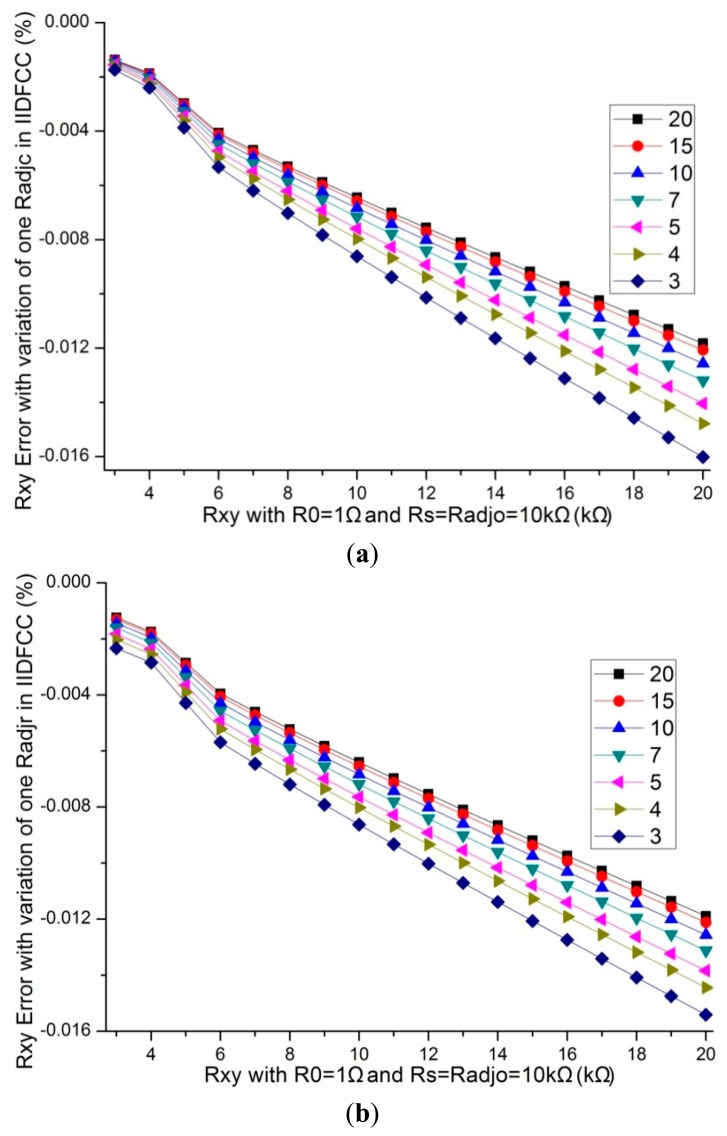
(**a**) The *R_xy_* errors of the variation of one *R_adjc_* in IIDFCC where *M* = *N* = 8, *R_0_* = 1 Ω and *R_adjo_* = 10 kΩ; (**b**) The *R_xy_* errors of the variation of one *R_adjr_* in IIDFCC where *M* = *N* = 8, *R_0_* = 1 Ω and *R_adjo_* = 10 kΩ.

All other elements in resistive sensor arrays affected the measurement error of the EBT, in which the adjacent elements played a significant role [15,16]. We fixed the resistance value of non-scanned elements and all other adjacent elements (*R_adjo_*) at 10 kΩ, *M* = *N*= 8, *R_0_* = 1 Ω, *R_s_* =10 kΩ, the resistance value of one adjacent row element (*R_adjr_*) or one adjacent column element (*R_adjc_*), and the resistance value of the EBT varied in 3 kΩ–20 kΩ, the simulation results of IDFC [18], IIDFC [21], and IIDFCC with NI Multisim were shown in Tables 1 and 2, and Figure 5.

From Tables 1 and 2, and Figure 5, we found that the *R_xy_* errors of IIDFCC were significantly smaller than those of IDFC and IIDFC; the adjacent row elements and the adjacent column elements had a similar influence on the *R_xy_* errors in IIDFCC; with a same variation of one adjacent row element or one adjacent column element, the *R_xy_* errors change of IIDFCC was the minimum one in these three circuits. Thus, IIDFCC has a better performance than IDFC and IIDFC for reducing the *R_xy_* errors caused by the adjacent column elements.

### 3.5. Comparison with Other Methods

**Figure 6.**
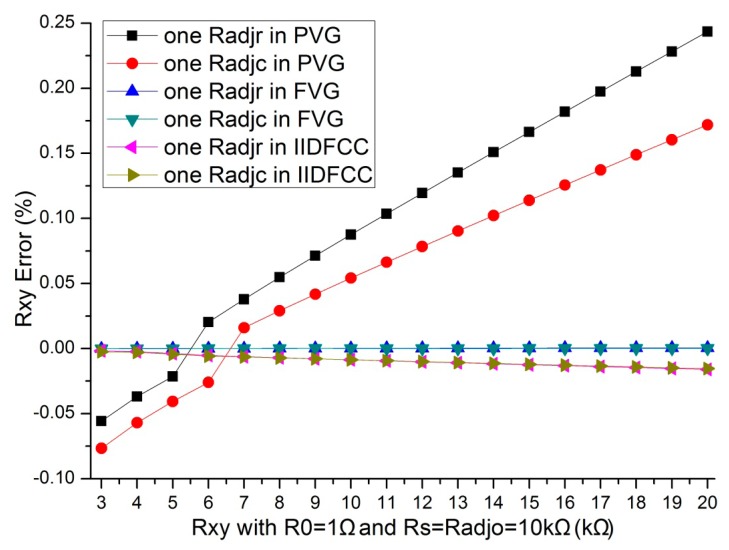
The *R_xy_* errors of the variation of one *R_adj_* where *M* = *N*= 8, *R_0_* = 1 Ω and *R_adjo_* = 10 kΩ.

In [5–7,19,20], the part virtual ground (PVG) technology was used. In [3,4,8,14], the full virtual ground (FVG) technology was used. Under the similar condition as Section 3.4, we implemented simulations for comparing the crosstalk suppression method of IIDFCC with PVG method and FVG method. The simulation results were shown in Figure 6.

From Figure 6, we found that, the *R_xy_* errors of the variation of one *R_adj_* were different in the three methods. The result of the method with PVG is the worst in the three methods. The crosstalk suppression method with IIDFCC is approximated to the crosstalk suppression method with FVG.

### 3.6. Discussion

Finally, Table 3 shows the comparison of the proposed method and other reported methods.

In our method, only one op-amp and *M* + *N* mux are used. So the method has the minimum complexity. For testing one element in the networked resistive sensor array, two analog-digital conversions are needed. So analog-digital conversions of 2(*M* × *N*) are necessary for scanning all elements in the networked resistive sensor array. Because the crosstalk caused by parasitic parallel paths for the multiplexers' channel resistor, the adjacent column elements, and the adjacent row elements were suppressed, this method has a good performance in the test accuracy of the EBT. So our method achieves a similar performance in the measurement accuracy as in the literature [3,4,8,14], while it has less complexity and less cost. In future work, we will implement a practical measurement on the proposed method.

## 4. Conclusion

Firstly, IIDFCC with one op-amp has been proposed to suppress the crosstalk for parasitic parallel paths in the 2-D resistive sensor arrays with the row–column fashion. Then, the equivalent resistance expression of the EBT in the networked resistive sensor array has been given. Subsequently, the effects of different parameters such as the multiplexer's channel resistors, the resistance range of the element being tested, the array size, and the adjacent elements on the measurement accuracy of the element being tested have been simulated with National Instrument Multisim 12. The simulation results show that, with our method, the resistance measurement error caused by the multiplexers' channel resistor, the array size, and parasitic parallel paths is reduced greatly. Furthermore, multiplexers with a bigger channel resistance can be used in the 2-D resistive sensor arrays.

## Figures and Tables

**Table 1.** *R_xy_* Error with one adjacent column element varied in 3–20 kΩ.

**Up Limited Error (%)**			**Low Limited Error (%)**			**Maximum Variation (%)**		
		
**IDFC**	**IIDFC**	**IIDFCC**	**IDFC**	**IIDFC**	**IIDFCC**	**IDFC**	**IIDFC**	**IIDFCC**
0.2255	0.1356	−0.0014	0.1126	0.0705	−0.0160	0.1129	0.0651	0.0146

**Table 2.** *R_xy_* Error with one adjacent row element varied in 3–20 kΩ.

**Up Limited Value (%)**			**Low Limited Value (%)**			**Maximum Variation (%)**		
		
**IDFC**	**IIDFC**	**IIDFCC**	**IDFC**	**IIDFC**	**IIDFCC**	**IDFC**	**IIDFC**	**IIDFCC**
0.2238	0.1587	−0.0012	0.1123	0.0653	−0.0154	0.1115	0.0933	0.0142

**Table 3.** Comparison of different implementations of the *M* × *N* networked resistive sensor array.

**Literature**	**Complexity**		**Error Source**		**Scanning Times**	**Accuarcy**

	**Nr. of Op-Amps**	**Nr. of Mux**	**Row or Column Adjacent Elements**	**Channel Resistor of Row or Column Mux**		
1,7,9,11,12,17,18	1	*M* + *N*	both	both	*M* × *N*	good
5–7,19,20	*N or M*	*M* + *N*	both	both	*M* or *N* with *N* or *M* ADC	good
21	1	*M* + *N*	row or column	row or column	*M* × *N*	better
proposed	1	*M* + *N*	none	none	2(*M* × *N*)	Much better
8,14	*M* + *N*	*M* + *N*	none	none	*M* × *N*	best
3,4	*M* + *N*	*M*	none	none	*M* with *N* ADC	best
